# Hyaluronan and Its Receptors: Key Mediators of Immune Cell Entry and Trafficking in the Lymphatic System

**DOI:** 10.3390/cells10082061

**Published:** 2021-08-12

**Authors:** Louise A. Johnson, David G. Jackson

**Affiliations:** MRC Human Immunology Unit, MRC Weatherall Institute of Molecular Medicine, University of Oxford, Oxford OX3 9DS, UK; louise.johnson@imm.ox.ac.uk

**Keywords:** lymphatic endothelium, hyaluronan, dendritic cell, T cell, macrophage, trafficking, immune cell, LYVE-1, CD44, glycocalyx

## Abstract

Entry to the afferent lymphatics marks the first committed step for immune cell migration from tissues to draining lymph nodes both for the generation of immune responses and for timely resolution of tissue inflammation. This critical process occurs primarily at specialised discontinuous junctions in initial lymphatic capillaries, directed by chemokines released from lymphatic endothelium and orchestrated by adhesion between lymphatic receptors and their immune cell ligands. Prominent amongst the latter is the large glycosaminoglycan hyaluronan (HA) that can form a bulky glycocalyx on the surface of certain tissue-migrating leucocytes and whose engagement with its key lymphatic receptor LYVE-1 mediates docking and entry of dendritic cells to afferent lymphatics. Here we outline the latest insights into the molecular mechanisms by which the HA glycocalyx together with LYVE-1 and the related leucocyte receptor CD44 co-operate in immune cell entry, and how the process is facilitated by the unusual character of LYVE-1 • HA-binding interactions. In addition, we describe how pro-inflammatory breakdown products of HA may also contribute to lymphatic entry by transducing signals through LYVE-1 for lymphangiogenesis and increased junctional permeability. Lastly, we outline some future perspectives and highlight the LYVE-1 • HA axis as a potential target for immunotherapy.

## 1. Introduction

The lymphatics play multiple roles in maintaining homeostasis in virtually all tissues through the uptake and drainage of interstitial fluids and the transport of electrolytes, dietary lipids, cholesterol, extracellular matrix and other dissolved macromolecules for appropriate degradation or delivery to the systemic blood circulation [1,2]. Although frequently overlooked by immunologists, the lymphatics are also integral to the immune system, insofar as they act as conduits for the migration of immune cells from tissues to draining lymph nodes (dLNs) for the generation, maintenance and modulation of antigen-specific immune responses [3,4,5,6,7], and later for the removal of phagocytes, apoptotic cell bodies and tissue debris during the repair of injured and inflamed tissues [8,9]. This continuous flow of information between tissues and downstream lymph nodes enables small numbers of patrolling antigen-presenting dendritic cells (DCs), recirculating memory T cells (T_RCM_) and regulatory T cells (T_REG_) to maintain constant surveillance for microbial or viral infection through a local neighbourhood watch system, licensed by their expression of CCR7, the receptor for the key chemokine CCL21 that directs lymphatic entry [10,11,12,13,14,15]. Moreover, in response to inflammation, the local release of cytokines such as IL-1, TNFα and INFγ induces large-scale mobilisation of resident DCs within tissues as well as an influx of T cells, monocytes and neutrophils from the circulation, all of which leads to a dramatic increase in immune cell trafficking through afferent lymphatics for the purpose of expanding and modifying immune responses in the dLNs [3,16,17,18]. Given the obvious importance of such trafficking for normal health and disease, there is a clear and present need to understand its underlying molecular mechanisms so that new and more effective therapies can be developed for lymphatic blockade in immune and inflammatory disorders.

Over the past two decades, a large number of different chemokines, adhesion receptors and ligands have been identified that each play supporting roles in immune cell entry and migration through afferent lymphatics, with many also expressed in the blood vasculature (see, e.g., [3,5,6,7,19,20] for reviews). In more recent years, evidence has emerged for an important involvement of the extracellular matrix glycosaminoglycan hyaluronan (HA) in lymphatic trafficking [21]. This negatively charged polysaccharide, which plays a well-documented role in both the extravasation of activated lymphocytes and neutrophils from inflamed blood capillaries via its primary receptor CD44, is now known to facilitate DC and macrophage entry to lymphatic capillaries, in association with its second receptor, the well-known and widely used lymphatic vessel marker LYVE-1 [6,22,23,24,25]. In this review, we present the current state of knowledge about HA and its receptors in lymphatic trafficking, with a particular emphasis on molecular mechanisms. Starting with a brief introduction to HA and its chief biological functions, we go on to describe how this ubiquitous polysaccharide and its complementary receptors LYVE-1 and CD44 come together to facilitate the entry of immune cells to afferent lymphatic capillaries. Additionally, we describe the novel and unusual character of LYVE-1 • HA interactions that support the process, as well as their significance for normal immunity, inflammation and disease, and the potential for HA and its receptors as targets for immune blockade.

## 2. The Multifunctional Polysaccharide Hyaluronan and its Intimate Association with Lymphatics

**Figure 1:**
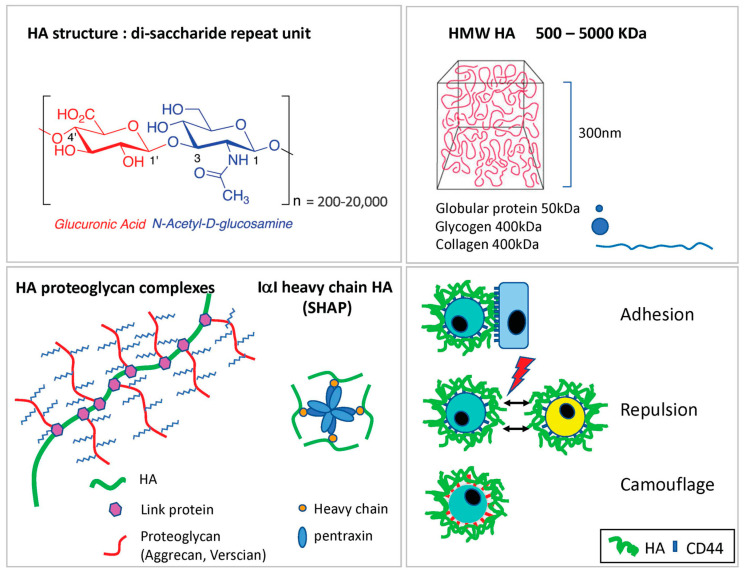
Structure and properties of HA. **Top left and right panels,** respectively, show the basic disaccharide repeat unit of long unbranched HA chains (represented in chair conformation) and the 3D volume occupied by a typical HMW HA chain (example shown is a 5000 kDa polymer) relative to average-sized globular proteins and larger globular/fibrous macromolecules depicted in 2D. **Bottom left panel** shows an illustration of a large noncovalent HMW HA complex containing the chondrotin sulphate proteoglycans aggrecan, versican and Link protein typical of those found in extracellular/pericellular matrices surrounding, e.g., chondrocytes in connective tissue anchored by CD44, and an inflammation-induced IαI heavy chain covalent HA complex (a.k.a. SHAP, serum HA associated protein) containing the cross-linking tetramer pentraxin. Formation of SHAP is catalysed by the small HA-binding protein TSG-6, which forms transient covalent intermediates with HA (not shown), but TSG-6 can also form cross-linked noncovalent complexes that have enhanced binding avidity for LYVE-1 and CD44 [49,50] (see text for details). **Bottom right panel** shows the different consequences of HA glycocalyx formation on cell behaviour including adhesion to neighbouring cells expressing appropriate HA receptors, repulsion by neighbouring cells with similar HA glycocalyces and camouflage of surface receptors and adhesion molecules depicted as red boxes, beneath the canopy formed by the HA glycocalyx.

HA, a large unbranched glycosaminoglycan of up to several mega Daltons in size is a co-polymer comprising multiple repeats of the disaccharide [*N*-acetyl glucosamine (β-1,4) glucuronic acid (β-1,3)]_n_, that forms a ubiquitous component of interstitial matrix (Figure 1) [21,26,27]. Amongst its many functions in soft tissues, HA provides structural support while at the same time maintaining a low resistance environment permissive for cell migration, a balancing act enabled by its capacity to absorb water equivalent to many times its mass and form hydrated gel-like meshworks that fill tissue spaces [26,27,28,29]. Synthesised by three distinct HA synthases (HAS1-3) located at the inner surface of the plasma membrane, each enzyme generates polymers of distinct size ranges (HAS1 and HAS3, 0.1–2 MDa; HAS2, >2 MDa) intrinsic to their structure and cellular regulation [30,31], by an unusual mechanism in which the nascent HA chains are simultaneously elongated and extruded to the cell surface [32,33]. The long chains emerge as free polymers, which subsequently form macromolecular complexes with one or more of the many known HA-binding partners that include the Link superfamily proteoglycans Aggrecan, Versican and Link protein, which together with type I collagen and fibronectin form cross-linked complexes in the pericellular or extracellular matrix of most organs [34,35]. Additionally, in inflammation, HA can form covalent adducts with the heavy chains of serum inter-alpha trypsin inhibitor IαI, in a trans-esterification reaction catalysed by the inflammation-associated TSG-6 (TNFα stimulated gene 6), and these may in turn be cross-linked noncovalently by tetrameric pentraxins (Figure 1) [36,37,38,39,40,41]. Such macromolecular complexes have been identified in appropriately activated vascular smooth muscle cells and kidney fibroblasts in vitro and in the vascular endothelium of injured liver and diabetic pancreas in vivo where they are thought to recruit immune cells through their heightened avidity for binding CD44 [42,43,44,45,46,47,48].

The extracellular matrix surrounding lymphatic vessels in most tissues is an HA-rich environment. Furthermore, the metabolism of HA within tissues is intimately associated with the lymphatics [21]. Notably in skin, HA undergoes a relatively rapid turnover (T_1/2_ ≥ 48 h) by cell-associated hyaluronidases, and the intermediate-sized cleavage products are carried away and transported in afferent lymph to dLNs [51], where they are terminally degraded through uptake by an endocytic HA receptor termed HA receptor for endocytosis (HARE), also known as Stabilin-2 in the LN sinus endothelium [52,53,54,55,56]. Importantly, in inflammation or injury, HA turnover is further increased and this generates lower molecular weight (<500 kDa) degradation fragments (LMW HA) that have been reported to act as danger signals, otherwise known as damage associated molecular patterns (DAMPS) or “Alarmins” through binding to the toll-like receptors TLR2 and TLR4, and the leucocyte HA receptor CD44 on innate immune cells [57,58,59,60]. Unlike intact high molecular weight HA (HMW HA), which is generally anti-inflammatory [61,62,63], these smaller fragments are widely believed to be drivers of inflammation that induce the production of cytokines, chemokines, matrix metalloproteases (MMPs) and nitric oxide from various cell types [64,65,66,67] as well as promoting both angiogenesis and lymphangiogenesis and increased lymphatic vessel permeability, as discussed further below [68,69,70]. The functional definition of low MW HA (generally 10–500 kDa) is somewhat vague and varies between authors. In addition, the issue of how cells discriminate low MW from high MW HA (generally > 1000 kDa) and respond accordingly is still contentious [71]. However, it is currently thought to be a consequence of polymer valency and the relative ability of the different sized polymer chains to induce receptor clustering and downstream signalling [23,72,73,74].

Arguably the most important function of HA is in support of cell:cell and cell:matrix adhesion during cell migration, achieved by means of stable and/or transient interactions with its receptors on neighbouring static and migratory cells respectively (see e.g., [75,76]). Integral to this is the capacity of HA to form a circumferential matrix or glycocalyx on the surface of appropriate cell types through retention of the polymer by its primary receptor CD44 in the plasma membrane [77,78,79,80]. Indeed, such a CD44-tethered HA glycocalyx on the luminal endothelial surface of post-capillary blood venules serves to capture circulating inflammatory cells, including T lymphocytes and neutrophils from flow via their own separate expression of CD44, thus facilitating their extravasation to the underlying tissue through formation of a CD44 • HA • CD44 sandwich [81,82,83,84,85,86].

## 3. Location of LYVE-1 in the Specialised Junctions of Initial Lymphatic Capillaries

**Figure 2:**
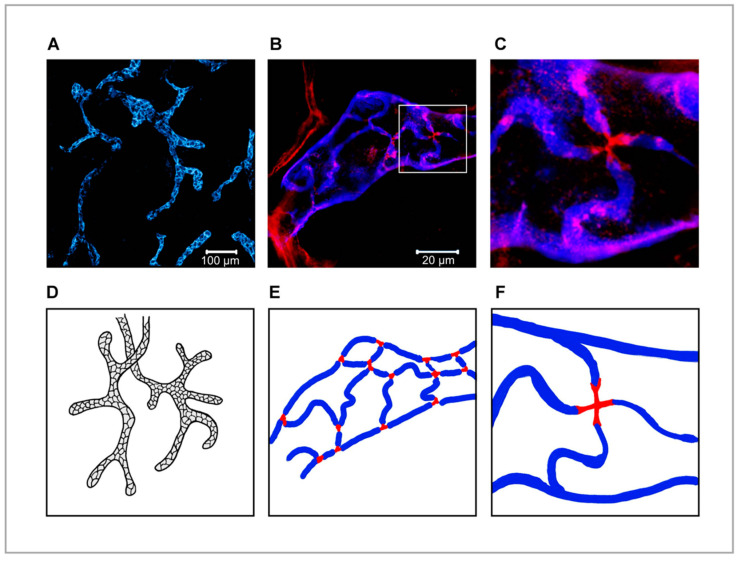
Functionally specialised junctions between endothelial cells of initial lymphatic vessels. (**A**–**C**), Blind-ended initial capillaries of whole mount mouse ear dermis, immunolabeled with anti-LYVE-1/AF647-conjugated anti-rabbit antibody (blue) and anti-VE-cadherin/AF568-conjugated anti-rat antibody (red) shown at low (100×, **panel A**) and high magnification (630×, **panel B**), with further digital zoom (**panel C**), to illustrate the arrangement of LYVE-1 along endothelial flaps pinned by VE-cadherin in button-like junctions where immune cells transmigrate (see text for details). The same images are re-drawn as illustration models in panels (**D**–**F**).

Seminal studies of immune cell trafficking via lymph, most notably the exit of DCs from the inflamed mouse trachea, have shown that migrating leucocytes commonly target the first few millimetres of the initial capillaries to enter afferent lymph and traffic to downstream LNs [87]. Unlike the more tightly sealed pre-collectors and valved collectors into which they drain, these blind-ended vessels have distinctive semi-permeable intercellular junctions, created by the interdigitation of their constituent oakleaf-shaped endothelial cells. This unusual architecture generates alternating flap-like portals of 0.5–1 μm diameter that are buttoned at their sides by a combination of the tight junction proteins ZO-1, ESAM and claudins and the adherens-junction protein VE-cadherin, and these are now recognised as dedicated points of cell entry [87,88,89]. Strikingly, it is along such flaps that the lymphatic endothelial HA receptor LYVE-1 [24] is concentrated (Figure 2), together with the homotypic adhesion receptor PECAM-1 (CD31), [87,88], while LYVE-1 is present at lower levels in pre-collectors and almost completely absent from the tightly zippered junctions of distal valved collectors [90,91]. As will be described in detail below, this discrete location positions the receptor optimally for its role in mediating the adhesion and entry of incoming leucocytes. In addition to such adhesion, the process of immune cell intravasation is driven by a combination of actomyosin-mediated immune motility and β2 integrin activation (according to inflammatory status), directed primarily by the chemokine CCL21, which is constitutively synthesised and secreted by LECs to form haptotactic gradients as well as localised puncta [92] that are detected by migrating DCs [93,94,95,96,97], recirculating memory T cells (T_RCM_), macrophages and some neutrophils [98] through the signalling receptor CCR7 [12,14,15]. Intriguingly, contact between transiting DCs and vessel endothelium can also trigger rapid “on demand” discharge of CCL21 from pre-stored depots in *trans*-Golgi vesicles to sites of entry on the basolateral surface of the endothelium by a Ca^2+^-triggered exocytic mechanism that involves transport by microtubules and fibrillar actin [99].

While the foregoing studies indicate immune cells enter lymphatics via overlapping “buttoned” junctions under resting conditions or during the initial acute response to inflammatory stimuli (i.e., brief exposure to LPS), it is now apparent from studies just published by the group of Cornelia Halin that leucocytes can also enter tightly zippered collector vessels that lack LYVE-1, during conditions of chronic inflammation [100]. This is consistent with the fact that newly sprouting lymphatic vessels generated by inflammation-induced lymphangiogenesis initially form such zippered junctions, like lymphatics in pre-term embryos [101,102]. Furthermore, exposure of existing lymphatic capillaries to inflammatory cytokines such as TNFα and IL-1 leads to the upregulated expression of ICAM-1, VCAM-1 and E-selectin—receptors that are known to mediate leucocyte transit across tight endothelial junctions in inflamed blood vessels and which were shown to mediate DC entry to lymphatic vessels in inflamed tissue via leucocyte β2 and β1 integrins respectively [103,104,105]. Use of this second entry route may well be necessary in inflammation when the rate of default entry through button junctions becomes limiting, and when induction of ICAM-1 and VCAM-1 is required for transit across zippered junctions in collector vessels.

## 4. Lymphatic Entry: The Immune Cell HA Glycocalyx and Its Engagement with LYVE-1

**Figure 3:**
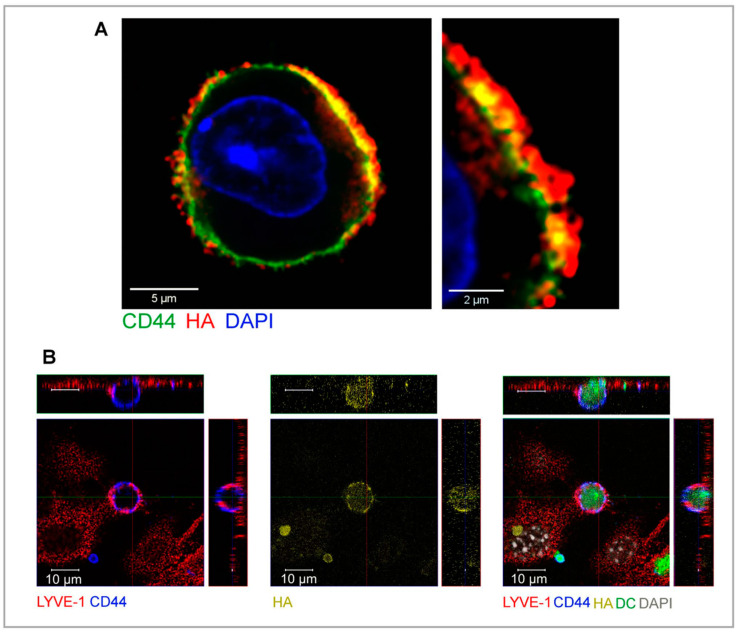
The HA glycocalyx on the surface of DCs and its involvement in docking to lymphatic endothelium via LYVE-1 transmigratory cups. Panel (**A**) shows the HA glycocalyx on the surface of an LPS-matured mouse bmDC, detected with bVG1/streptavidin-AF647, dual stained for CD44/AF488 and counterstained for nuclei with DAPI, imaged by confocal microscopy with Airyscan detection and digital zoom (right). Panel (**B**) illustrates the docking of a mouse bmDC to the surface of a LEC through formation of a LYVE-1 transmigratory cup that encircles the cell and engages with the HA glycocalyx. Shown are confocal microscopy images with orthogonal views of mouse LEC monolayers immunostained with anti-LYVE-1/AF548 (red), recorded 3 h after the addition of LPS-matured fluorescent bmDCs (green) dual immunostained for anti-CD44/AF594 (blue), and bVG1/streptavidin-AF647 (yellow), and counterstained for nuclei with DAPI (grey). Adapted from Johnson et al. 2021 [114] under Creative Commons BY 4.0.

It was originally envisaged that LYVE-1 would function in an analogous manner to its homologue CD44 in blood vessel endothelium and mediate immune cell entry by sequestering a boundary layer of hyaluronan to which the migrating immune cells could adhere (via leucocyte CD44) for transmigration to the lumen [6,24,25,106,107]. However, it is clear from recent and ongoing studies that in lymphatic migration, immune cells themselves provide the hyaluronan for LYVE-1 mediated adhesion, through their ability to synthesise and assemble a HA glycocalyx on their exterior surface [22,49]. Although the capacity of certain immune cell populations for HA synthesis had been reported previously by other workers [108,109,110,111,112,113], the key significance of the glycosaminoglycan for lymphatic entry and migration was completely overlooked. Generated primarily by the hyaluronan synthase II isoenzyme that makes polymer chains of up to 10,000 saccharide units in length (equivalent to contour lengths of several microns), the HA glycocalyx of human and murine monocyte-derived dendritic cells and their endogenous counterparts in tissue constitutes a dense corona of some 500 nm thickness, as estimated by conventional and high-resolution Airyscan confocal imaging using either biotinylated HA-binding protein (bHABP) or biotinylated versican G1 domain (bVG1) and fluorescent streptavidin as probes (Figure 3) [114]. The constituent HA polymers of the glycocalyx are, in turn, anchored to the plasma membrane via CD44, as deduced from analyses of DCs isolated from receptor knockout mice, which indicated the *cd44^−/−^* cells continue to synthesise HA but fail to retain a glycocalyx on their surface. Indeed these studies showed the nascent HA chains are exported to the surface along with CD44 in intracellular vesicles, suggesting that glycocalyx assembly and receptor biosynthesis are linked and may well be co-ordinately regulated [114].

Direct evidence that LYVE-1 • HA glycocalyx interactions are critically important in vivo for immune cell docking and entry to initial lymphatics vessel entry came from comprehensive analyses of DC trafficking in wild-type and *Lyve1^−/−^* mice in an oxazolone induced model of skin contact hypersensitivity (CHS) using FITC skin painting [22]. These revealed a marked delay and transient reduction in the numbers of endogenous CD11c^+^ dermal DCs recovered from the skin draining cervical LNs of *Lyve1^−/−^* animals 24 h after oxazolone sensitisation, which was shown to result from log-jamming at the basolateral surface of initial capillaries in experiments with dermally injected CMFDA-labelled mouse bone marrow DCs (bmDCs) imaged over corresponding time-points by confocal microscopy. Similar studies comparing the migration of co-injected control and hyaluronidase-treated DCs, and co-injected *cd44*^−/−^ and wild-type littermate DCs, fully corroborated these findings, emphasising the importance of CD44 and the HA glycocalyx for LYVE-1 mediated adhesion and entry via these dermal lymphatics [22,114]. Furthermore, lymphatic entry and LN trafficking of both endogenous CD11c^+^ DCs and adoptively transferred bmDCs were also impeded in the CHS model by co-administration of either LYVE-1 or CD44 HA-blocking mAbs, and this was again confirmed in ex vivo crawl-out assays with dorsal ear skin [22]. While yet to be confirmed in live tissues or explants by real-time video microscopy, engagement of migrating DCs with LYVE-1 in the endothelial junctions of initial capillaries likely leads to their transient unbuttoning (i.e., disruption of VE-cadherin homotypic adhesion complexes, claudins, ZO-1 and ESAM) to create appropriately expanded portals for entry. It was assumed during these earlier studies that the delay rather than sustained blockade in trafficking of DCs to dLNs in *Lyve1^−/−^* mice and its recovery at later time-points were due to compensatory gene expression during embryonic development [22]. However, in light of the recent finding that DCs can also enter the lymphatics through the conventional zippered junctions of LYVE-1^-^^ve^ downstream collectors by an additional, slower mechanism involving β1 integrin/VCAM-1 mediated adhesion, this seems the more likely explanation [100].

Lastly, the significance of LYVE-1 • HA-mediated DC migration for in vivo immune function is underscored by experiments in which mice were immunised intradermally with free ovalbumin or ovalbumin peptide-loaded DCs and assessed for the effects of LYVE-1 disruption on subsequent *ova*-specific T cell responses in dLNs [22]. These showed that both *Lyve1* gene deletion and mAb blockade disrupted the generation of ova-specific CD4 and CD8 T cell proliferative responses in downstream lymph nodes, confirming that in vivo, the process of LYVE-1 • HA-mediated lymphatic entry can be rate-limiting for protective immunity [22].

## 5. Transmigratory Cups—How Docking of the HA Glycocalyx with LYVE-1 Enables Immune Cell Transit to the Vessel Lumen

While the various animal studies described above have affirmed the physiological importance of HA and its receptors for immune cell docking and entry to the lymphatics, elucidating the underlying molecular mechanisms has largely involved studies of immature and LPS-matured human and murine monocyte-derived DCs adhering to and transmigrating monolayers of primary dermal lymphatic endothelial cells (LECs) in vitro. These revealed that within minutes of initial physical contact, each individual DC induces the formation of a LYVE-1-enriched ring-like transmigratory apparatus in the underlying LEC monolayer. Viewed orthogonally and in z-sections by confocal microscopy, these structures appear in vitro as cup-like protrusions that extend towards individual adherent DCs and gradually envelop them as they begin to transit across the endothelium (Figure 3) [22]. Given the term LYVE-1 “transmigratory cups” owing to their resemblance to the characteristic ICAM-1 and VCAM-1 enriched membrane protrusions that form in vascular endothelium during lymphocyte transit [115,116,117,118], and because they appear to be essential for DC traversal, these lymphatic endothelial docking structures are critically dependent on the integrity of all three components, LYVE-1, CD44 and HA, for their assembly [114]. Importantly, these docking structures are also observed in vivo in mouse skin afferent lymphatic capillaries during adhesion and transit of endogenous DCs [22].

**Figure 4:**
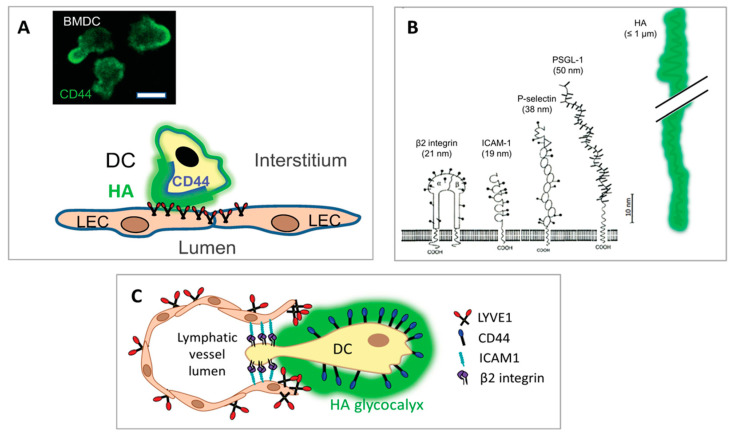
Dynamics of the DC HA glycocalyx and its influence on engagement of underlying adhesion receptors with lymphatic endothelium during vessel entry. (**A**) Initial contact with lymphatic endothelium triggers polarisation of migrating DCs and redistribution of CD44 to the adhesive uropod as observed by confocal microscopy [114] and as depicted in the illustration shown. Bar = 10 µm. (**B**) The estimated length of HMW HA chains in the glycocalyx (≤1 µm) extends beyond that of other key underlying adhesion receptors such as leucocyte integrins and their ligands on the DC surface, likely positioning the LYVE-1 • HA axis for making the first adhesive contacts between DC and endothelium during the process of transmigration. (**C**) Speculative organisation of DC • endothelial contacts during transmigration and the possible influence of the HA glycocalyx on underlying β2 integrin accessibility and adhesive function. The illustration depicts the possible imposition of focal clustering of DC integrins by the bulky HA chains, enabling their conformational activation and subsequent contribution to endothelial adhesion via ICAM-1 during diapedesis [128]. How and when integrins become accessible beneath the glycocalyx is, however, still unclear. The confocal image in panel A is from Johnson et al. 2021, [114] and the cartoon in panel B is adapted from Barclay et al. 1997, [129] with permission from the publisher (Elsevier).

Our recent studies into the dynamics of DC:LEC adhesion using video microscopy have revealed the migrating cells first attach to the LYVE-1 transmigratory cups via their posterior pole or uropod, before extending their leading edge or lamellipodium to explore the endothelial surface for nearby junctions where they subsequently transit [114]. Notably, CD44, which is known to be important for formation of this dynamic foot-like protrusion in motile T cells and neutrophils [86,119,120,121] and which we showed to be essential for both DC adhesion and lymphatic transmigration, localises almost exclusively to the DC uropod [114]. Here, the receptor likely distributes the HA glycocalyx for optimal engagement with LYVE-1 and LEC transmigratory cup formation (Figure 4A), driven by polar re-arrangement of the cortical actin cytoskeleton to which CD44 is anchored via ERM proteins [122,123,124,125]. Later, disengagement of the DC during transendothelial transit appears not to require shedding of LYVE-1 or the CD44-bound glycocalyx (Johnson, L. A. and Jackson, D. G., unpublished), despite the fact the former can be achieved in principle by ADAM 17 and MT1-MMP-catalysed cleavage at discrete sites (F226-E229 and A235-L236, respectively) close to the LYVE-1 transmembrane anchor under appropriate circumstances [126,127]. Rather, the unusual nature of LYVE-1 • HA interaction may allow such detachment to be accomplished by tractive forces generated by the DC itself or even interstitial flow (see below).

**Figure 5:**
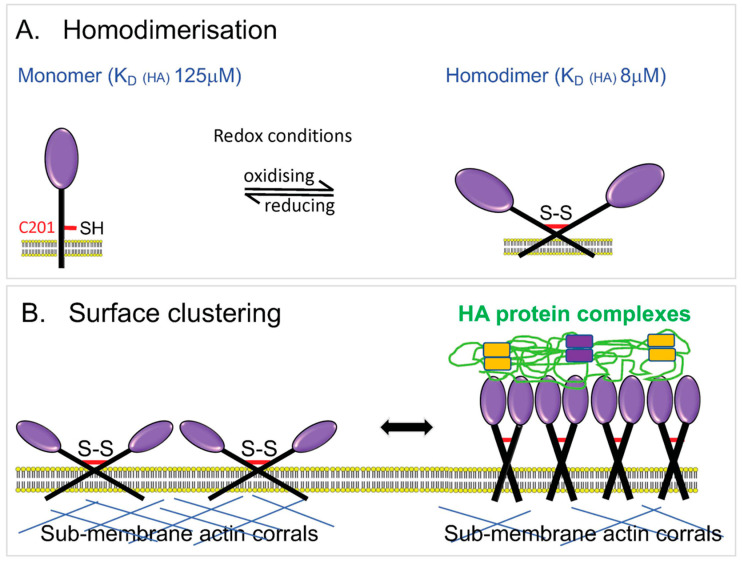
Characteristics of LYVE-1 that regulate its interactions with HA in lymphatic endothelium. (**A**) Endogenous LYVE-1 forms homodimers on the endothelial surface via an unpaired cysteine residue C201 in a process that is critical for HA-binding in vivo and which increases binding affinity some 15-fold as assessed by Biacore analysis [147]. The ratio of homodimer to monomer is subject to regulation by the local redox environment. (**B**) Engagement with HA also requires clustering of LYVE-1 on the endothelial surface, induced by interaction with cross-linked macromolecular HA • protein complexes that have heightened binding avidity (see also Figure 1), and local disassembly of the sub-membrane actin meshwork that normally constrains LYVE-1 lateral mobility in the plasma membrane [49,150]. The figure is modified from Jackson (2019) [23] with permission from the publisher (Elsevier).

Although yet to be formally demonstrated for DCs in vivo, it is also likely that the adhesivity of the glycocalyx for LYVE-1 may be influenced by the incorporation of additional HA-binding proteins into its structure. Notably, TSG-6, the matrix proteoglycan versican, the inter-alpha trypsin inhibitor (IαI) heavy chain and the cross-linking pentraxins that are each synthesised by leucocytes of the myeloid lineage in response to inflammation [130,131], have the capacity to form complexes with HA and dramatically enhance binding to its receptors [39,42,44,50,130,131] (see Figure 1 and Figure 5). Furthermore, the amount of HA incorporated into the DC glycocalyx and its surface density may be influenced in vivo by cytokines such as TNFα, IL-1 and IFN-γ that are known to promote both HA synthesis and CD44-binding affinity in different cellular contexts [132,133,134,135,136] and which may thus alter the strength and duration of LYVE-1 • HA glycocalyx interactions. Indeed, this same process occurs in vitro as in the case of IFN-γ and LPS during maturation of bmDCs and macrophages [22,114]. As a further proof of principle, we recently showed that augmenting CD44 HA-binding by in vitro treatment of bmDCs with the CD44 cross-linking mAb IRAWB14 dramatically increased the density of their HA surface glycocalyx and boosted their adhesion to LECs to such an extent that it blocked the subsequent ability of the DCs to transmigrate, and this was confirmed in vivo in the CHS model where the antibody markedly reduced DC migration to skin dLNs [114]. Of note, we found in the same study that bmDCs secrete up to 50% of their total cellular HA and hence they appear to carry sufficient spare capacity for additional incorporation of the glycosaminoglycan into the glycocalyx in response to such changes in their tissue environment [114]. Clearly, under physiological conditions, the rate and timing of LYVE-1-mediated DC trafficking could be finely tuned by the degree of inflammation or tissue injury.

## 6. Novel Structural and Biophysical Characteristics of the LYVE-1 HA Interaction That Facilitate Immune Cell Transit

The prominent role played by HA in immune cell adhesion and transit across lymphatic endothelium is facilitated by some unique physical characteristics of the polymer and its seemingly unique mode of interaction with its primary lymphatic receptor LYVE-1. Firstly, the large dimensions of HA molecules (with contour lengths of up to several microns) and their polyanionic nature, bestow the immune cell glycocalyx with the capacity to form a charged shell that extends far beyond the much smaller underlying adhesion receptors on the leucocyte surface such as β2 integrins that have ectodomain sizes in the region of 20 nm [23] (Figure 4B). This leads the glycocalyx to make the first adhesive contact between the migrating immune cell and the lymphatic endothelium through its receptor LYVE-1, whose relatively weak and reversible HA-binding characteristics (as discussed below), may help leucocytes avoid engaging their firm adhesion molecules (i.e., β1 and β2 integrins) in the early stages of diapedesis [22,23] (Figure 4C). Indeed, the comparative softness of the HA glycocalyx may well enhance the accessibility of these and other adhesion molecules on both the endothelium and immune cell surface, including such examples as CD137/4-1BB [137], Mannose receptor [138,139], CLEVER-1/Stabilin-1 [140,141], ALCAM [142], CD31 and CD99 [143], that are variously implicated in lymphatic entry and trafficking, as the brush-like border formed by the HA chains has been observed to indent upon engagement with its receptors [144]. Additionally, as shown by studies on tumour cells, the bulky nature of the HA glycocalyx may facilitate adhesion to underlying leucocyte integrins through their confinement within focal clusters and their conformational activation through tensile stress [128]. Hence it is evident that the glycocalyx facilitates immune cell adhesion and transmigration at least in part through its innate physical properties.

As regards the nature of this adhesion, LYVE-1, in common with CD44 [145], engages a short tract (minimal footprint size ≥ 8 sugar units) within the long HA polymer with relatively low monomer-binding affinity (125 μM) [146], and hence the capture of individual long chains on the LEC surface depends on simultaneous interactions with multiple adjacent receptor molecules to generate the necessary avidity for tethering [23]. Curiously, LYVE-1 forms homodimers on the LEC surface both in vitro and in vivo by means of a single conserved redox-sensitive intermolecular disulphide (C201) in its membrane-proximal domain, an association that more than doubles the HA footprint size to a 22 mer and increases HA-binding affinity some 15-fold, from 125 μM to 8 μM (Figure 5) [147]. Even so, homodimerisation is not sufficient in itself to enable constitutive binding of free HMW HA by the native receptor in LECs [148], and such binding requires further high-density clustering of LYVE-1 or prior cross-linking of HA in the form of multimolecular complexes with CD44 and/or one or more of its HA-binding partners [49]. This constraint is imposed by the limited lateral mobility of LYVE-1, owing to its corralling within the sub-membrane actin meshwork by means of the cytoplasmic tail, as evidenced by super-resolution microscopy [149,150]. As outlined earlier, the necessary conditions for HA-binding are met during DC transit by the redistribution of LYVE-1 from its normally punctate pattern in the plasma membrane [149] into receptor-dense lymphatic transmigratory cups at the leucocyte:endothelial interface, and by recruitment of the multivalent CD44 • HA glycocalyx to the pro-adhesive DC uropod [22,114]. Both these processes involve regulated assembly/disassembly of the sub-membrane actin cytoskeleton that restricts the membrane mobility of both LYVE-1 and CD44 [124,125,150]. Overall, it is likely that these features allow lymphatic vessels to discriminate between free HA present in interstitial fluid and cell (i.e., glycocalyx) bound HA and to respond appropriately to each.

**Figure 6:**
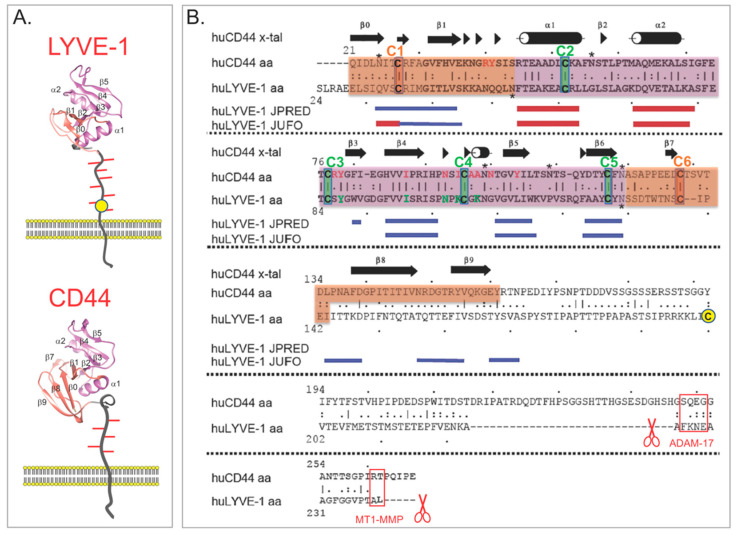
3D structure of the LYVE-1 HA-binding Link domain. (**A**) Structure-based models of the HA-binding domain in human LYVE-1 based on the high-resolution crystal structure of its closest homologue CD44 [152] below. Illustrations depict membrane-proximal domains in each case decorated with O-linked sugars (red lines). The yellow circle in LYVE-1 represents the unpaired cysteine residue C201 involved in homodimer formation (see Figure 5). (**B**) Alignment of the human LYVE-1 and CD44 amino acid sequences highlighting the regions encoding the conserved disulphide-bridged α/β fold of the consensus Link domain (mauve) and the β stranded *N*- and *C*-terminal extensions peculiar to CD44 (orange) stabilised by the additional disulphides C1-C6 and C2-C5 predicted to be present also in LYVE-1 from secondary structure program analysis using JPRED and JUFO. Notably in LYVE-1, the β8 and β9 strands of the C-terminal extension are substituted by serine and threonine-rich tracts that predict formation of an elongated, *O*-glycosylated membrane-proximal stalk [146]. Asterisks mark amino acid motifs (NXS/T) for N-glycosylation. Sites for LYVE-1 ectodomain cleavage (scissors symbol) by MMPs and ADAMs are boxed in red. The figure is modified from Jackson (2019) [23] with permission from the publisher (Elsevier).

The fine details of the HA-binding interaction in LYVE-1 are currently emerging from ongoing structural and biophysical studies of the receptor. As deduced from initial sequence comparisons, LYVE-1 is a classical type I transmembrane protein of approximately 60 kDa MW, closely related to the leucocyte receptor CD44 (45% sequence similarity) and bears a conserved disulphide-bonded HA-binding domain termed the Link module at the *N*-terminal end of a 211–212 residue extracellular domain, a 21 residue transmembrane anchor and a 63 residue cytoplasmic tail (Figure 6) [23,24,151]. In LYVE-1, as in CD44, the beta-fold of the consensus Link module is extended at both its *N*- and *C*-termini by three further β strands (β0, β1 and β7, respectively) and a third intramolecular disulphide bridge not found in any other members of the Link protein superfamily [23,145,146,152]. Notably, however, LYVE-1 lacks the additional *C*-terminal β8/β9 strands present in the CD44 Link module that are thought to mediate a mechanosensitive conformational switch between low and high affinity-binding states in transmigrating lymphocytes activated by high shear blood flow [146,153], likely reflecting the redundancy of this feature for immune cells migrating in the low shear environment of the lymphatic compartment.

More recent studies using X-ray crystallography have revealed that the HA-binding site in LYVE-1 occupies a deep cleft on one face of the Link module and that HA threads through one end of this cleft using a mixture of direct and water-mediated H-bonds to bind its surface (Banerji, Ni, Gilbert and Jackson, unpublished). Moreover, quantitative analysis of the binding mechanics at the single-molecule level by means of dynamic force spectroscopy, using immobilised recombinant human LYVE-1 and biotinylated HA (bHA), indicates the receptor has a marked preference for the free-ends of HA chains rather than internal regions, and that the individual binding interactions rupture collectively in response to longitudinal force, rather than sequentially as in the “bond-by-bond” breakage observed for CD44 [154,155]. These curious properties imply an unusual “sliding” interaction between HA and LYVE-1 rather than the firm “sticking” interaction mediated by CD44. Furthermore, the ready detachment of bound HA chains by pulling forces (~30 pN) in the range of those exerted during actin cytoskeleton-based cell motility and the low shear stresses of peri-lymphatic interstitial flow (<5 μN/cm^2^) appear to tune the LYVE-1 • HA interaction perfectly to support the chemokine directed inward crawling of immune cells into lymphatic capillaries (see also [23]).

## 7. Significance of the HA Glycocalyx in Different Immune Cell Populations: Macrophages, T Cells and Neutrophils

Not all immune cell populations utilise an HA glycocalyx to enter and migrate through lymphatics. Besides DCs, the other immune cells so far known to do so are macrophages [49,156]. These phagocytes, which comprise populations from two separate developmental origins, namely circulating bone marrow-derived monocytes that enter the tissues in response to inflammation (CX_3_CR1^int^Ly6C^+^/CX_3_CR1^hi^Ly6C^−^ cells in mice and CD14^+^CD16^+^/CD14^+^CD16^−^ cells in humans [157,158]), and self-renewing tissue-resident macrophages that derive from embryonic yolk sac [159,160], have been observed to exit tissue via afferent lymphatics during the later resolution phase of inflammation [8,9]. Both human and murine monocyte-derived macrophages can synthesise HA, and this has been shown to increase during their in vitro LPS/interferon-induced differentiation to an inflammatory M1-like phenotype, similar to that in inflamed tissues [130,131]. Moreover, as revealed initially by in vitro studies, such macrophages can assemble an endogenous HA glycocalyx with which they adhere and transmigrate lymphatic endothelium in a LYVE-1-dependent manner, as confirmed from experimental disruption of the process by LYVE-1 blocking mAbs and hyaluronidase digestion [49]. Moreover, the importance of this LYVE-1 • HA axis for in vivo macrophage trafficking was recently demonstrated in a mouse model of myocardial infarction (MI), induced by ligation of the coronary artery [161]. In this disease, which is characterised by oedema, neutrophil infiltration, massive cardiomyocyte death and the subsequent development of non-contractile cardiac scar tissue, M1 macrophages play a pivotal role in re-modelling the injured tissue through timely removal of apoptotic cells (efferocytosis), and secretion of cytokines and proteases, before differentiating to a reparative, anti-inflammatory M2 phenotype that is finally cleared from the myocardium [162,163,164,165]. Importantly, the mouse MI studies demonstrated that the recruited macrophages exit the infarcted myocardium to draining peri-aortic and paratracheal mediastinal nodes via the cardiac lymphatics and that the process is attenuated in *Lyve1^−/−^* animals, resulting in macrophage accumulation, aggravated fibrosis and delayed cardiac repair [161]. Furthermore, macrophage clearance was greatly enhanced by stimulating local lymphangiogenesis through administration of rVEGF-C156S, a process previously shown to reduce myocardial oedema and improve systolic function [166,167,168,169], and this clearance was again blocked in *Lyve1* knockout mice. Similar involvement of the HA glycocalyx and LYVE-1 is implicated for clearance of M1 macrophages from the inflamed peritoneal cavity to draining mediastinal (para-aortic) lymph nodes above the diaphragm, as evidenced from preliminary studies in *Lyve1^−/−^* mice using a model of sterile peritonitis elicited by Biogel^®^ bead injection ([156]; Bhattacharjee, S. and Jackson, D.G., unpublished).

The situation is still unclear for recirculating CD4 and CD8 T cells (T_RCM_), regulatory T cells (T_REG_), plasma cells and certain classes of innate lymphoid cells (ILCs) that exit from peripheral tissues via lymph in inflammation [19]. T cells, which are the most abundant migrating leucocytes in afferent lymph, express transcripts for HA synthases, and can both synthesise and secrete HA [108]. Furthermore, synthesis via HAS 1 and HAS 2 was recently shown to be essential for stabilising T cell • DC adhesion and immune synapse formation, as well as the priming and Th1 polarisation of peptide-specific T cell responses in mice [111,112,170]. Although the HA mediating these effects appeared to derive from DCs rather than T cells, more recent studies have revealed that HA synthesised by T cells also plays an important contributory role [171]. Whether an endogenous HA glycocalyx mediates T cell entry and trafficking in lymph, however, remains to be established. Curiously, though CD44 on the surface of T cells was reported to mediate lymphatic entry, this was not by adhesion to its ligand HA, but rather to the macrophage mannose receptor (MMR) present on lymphatic endothelium, which binds to covalently attached CD44 sugar chains [172,173]. In addition, the Link superfamily receptor CLEVER-1 (a.k.a. Stabilin-1, FEL1) expressed in both afferent and efferent lymphatics has also been shown to support T cell entry, but as its Link module does not support HA-binding, the identity of its ligand on T cells is unclear [174]. Moreover, T_EFF_ cells and T_REG_ enter the lymphatics by means of the β2 integrin • ICAM-1 and β1 integrin • VCAM-1 axes, directed by gradients of CCL21 and the chemoattractant S1P, the latter integrated by lymphotoxin (LTα1β3) and its receptor LTβR [175]. Hence, firm conclusions regarding the extent, if any, to which these key cells of the adaptive immune system use the LYVE-1 HA axis for lymphatic entry in vivo must await the outcome of further investigations.

Lastly, it is now evident that neutrophils neither biosynthesise HA nor assemble a surface HA glycocalyx [17]. These normally short-lived innate immune cells, which constitute the frontline defence against host tissue injury and infection, are recruited to tissues in response to inflammation. There, upon exposure to appropriate inflammatory cytokines, neutrophils evade apoptosis [176,177,178], exit tissues via afferent lymphatics and can transport phagocytosed micro-organisms to dLNs for antigen presentation and immune manipulation [16,179,180]. Intriguingly, and unlike DCs and macrophages, neutrophils enter the lymphatics by a complex mechanism that involves sequential adhesion to endothelium via β2 integrins, digestion of the basement membrane by MMPs and the serine protease elastase, and retraction of inter-endothelial junctions triggered by release of the chemorepulsive lipoxin 12-S-hydroxyeicosatetraenoic acid (12-S HETE), a metabolite of arachidonic acid generated by lipoxygenase action [17,181]. Whether this novel mechanism has evolved to enable rapid entry through the zippered junctions of inflamed collector vessels, or operates equally in buttoned initial capillaries is not yet clear.

In summary, therefore, the assembly of an HA glycocalyx and the use of HA-mediated mechanisms for lymphatic entry appear to be more characteristic of resident tissue-migrating leucocytes than those transient populations that enter from the circulation. It is tempting to speculate that the glycocalyx licenses such cells for selective entry via the LYVE-1^+ve^ buttoned junctions in initial capillaries, perhaps enabling them to inter-communicate in the capillary lumen, or even re-exit to the tissues.

## 8. Involvement of HA and LYVE-1 in Regulating Lymphangiogenesis and Junctional Permeability

Besides mediating immune cell entry to the lymphatics through assembly of a surface glycocalyx, HA in the tissue extracellular matrix undergoes increased turnover in response to inflammation, and its degradation products can potentially influence immune cell trafficking in such important ways as triggering increased vessel permeability and stimulating new lymphatic vessel growth. It is well documented that short chain HA fragments act as DAMPs that trigger pro-inflammatory responses such as the activation of macrophages and DCs and upregulated transcription of chemokines, cytokines and matrix metalloproteinases by means of Toll-like receptors and CD44 (reviewed in [70]). Furthermore, in the blood vasculature, there is extensive evidence that they promote endothelial proliferation (angiogenesis) and junctional permeability via CD44 and downstream activation of Src and ERK and various tyrosine kinase linked growth factor receptors [68,69,182,183,184,185,186,187,188].

Despite the fact only limited studies have been carried out to date on such responses in lymphatics, mostly in the context of tumour metastasis rather than immune cell trafficking and with transformed tumour-derived endothelia rather than authentic LECs, it is evident that low molecular weight HA (LMW HA) can exert similar effects in this vasculature. Initially, LMW HA (but not HMW HA) was shown to induce junctional retraction in monolayers of the lymphatic-like endothelial cell line SVEC 4.10 by binding to LYVE-1 and transducing intracellular signalling via MAP kinase/ERK, and Src for VE-cadherin phosphorylation and proteolytic degradation [189,190]. Curiously, these effects were also found to involve co-operative signalling via the sphingosine-1 phosphate receptor S1P3, which was upregulated by LMW HA, and activation of the Rho A GTPase Rac that is involved in actin assembly in lamellipodia formation [191]. These findings were subsequently confirmed in authentic primary human dermal LECs, and in a mouse melanoma metastasis model in vivo, which also revealed a further link between LMW HA-induced endothelial junctional opening and intracellular Ca^2+^ signalling [192]. Confirmation that LYVE-1 was responsible for these events was provided by inclusion of shRNA knockdown controls, largely excluding the possibility of Toll-like receptor activation by HA or by endotoxins that can be present as contaminants in incompletely purified preparations of the glycosaminoglycan.

Interestingly, however, LYVE-1 is not in itself a signalling receptor. While the 63 residue LYVE-1 cytoplasmic tail contains a small number of serine and tyrosine residues, these do not undergo phosphorylation either constitutively or in response to HA-binding [24,25]. Instead LYVE-1 appears to signal indirectly through the tyrosine kinase Src and crosstalk with growth factor receptor tyrosine kinase linked receptors. In support of this conclusion, LMW HA-induced VE-cadherin phosphorylation and subsequent LEC junctional relaxation can be blocked by selective inhibitors of epidermal growth factor receptor and VEGF receptor tyrosine kinases as well as specific inhibitors of Src kinases (Wang, Y.-J. and Jackson, D.G., unpublished). Furthermore, co-immune precipitation studies have indicated LYVE-1 physically associates with VEGFR and PDGFR in the LEC plasma membrane [189]. The mechanistic basis for selective signalling by LMW HA as opposed to HMW HA is currently not understood, but is thought to reflect either the inability of the former to adequately cross-link LYVE-1 in the plasma membrane or its ability to disrupt pre-formed LYVE-1 clusters. Nevertheless, recent in vitro studies in our own laboratory indicate the situation is more complex and that HMW HA can in fact induce junctional retraction in HDLECs through VE-cadherin disassembly, but in a transient (i.e., minutes) rather than a sustained manner (Wang, Y.-J. and Jackson, D.G., unpublished). Hence it is tempting to speculate that docking of immune cells to lymphatic endothelium via the HA glycocalyx and LYVE-1 may transduce signals for temporary junctional opening as part of the mechanism for lymphatic entry.

Finally, and again consistent with its production in inflammation, there is a small but growing body of evidence that LMW HA can also promote lymphangiogenesis. This process which is critical during embryonic development, is characteristically initiated in adults in response to tissue injury by the vascular endothelial growth factors VEGF-A, C and D through their receptors VEGFR2/3, the β1 integrins and Neuropilin 2 (NRP-2), as well as the angiopoietin (Ang1/2)/Tie1/2 axis (see [193,194,195] for reviews), wherein the newly sprouted lymphatic vessels play a vital role in the clearance of immune cells during the resolution of inflammation. The first reported involvement of HA in lymphangiogenesis came from in vitro studies with the mouse LEC-like cell line SVEC4-10, which indicated that HA 4–10 mer oligosaccharides induce endothelial proliferation, migration and tube formation in Matrigel via LYVE-1, in association with disassembly and re-assembly of the cortical actin cytoskeleton and activation of the mitogenesis-associated MAPK/ERK, protein kinase-c (PKCα/βII) and Sphingosine 1 phosphate (S1P1/Edg -3) signalling pathways [191,196]. In common with the effects on junctional permeability [192,197], these responses were specific to LMW HA and not shared by HMW HA. Subsequent studies confirmed the potency of LMW HA as a mitogen both for cultured HDLECs (2–7-fold increase in proliferation) and endogenous lymphatic capillaries in mouse skin explants [198]. In addition, they demonstrated that intradermal injection of mice with purified 8–50 mer HA oligosaccharide preparations of similar concentration to those found in tumours (1–10 μg/mL, equivalent to low mM) induced lymphangiogenesis directly via LYVE-1, as well as synergistically with VEGF-C but independently of CD44 or TLR4 [198]. Curiously, LMW HA was also found to upregulate synthesis of TGFβ by LECs, a cytokine that, in turn, downregulates LYVE-1 expression, indicating a possible feedback mechanism by which the glycosaminoglycan might limit the extent of lymphangiogenesis in vivo. Given the potency of LMW HA for inducing both LYVE-1 mediated lymphangiogenesis and junctional relaxation in the face of its low receptor-binding affinity (K_D_ 125 μM), it appears that lymphatic endothelium is extremely sensitive to this pro-inflammatory form of the glycosaminoglycan and that occupancy of only a small proportion or a discrete subpopulation of LYVE-1 molecules in the LEC plasma membrane is required to trigger these responses. Further, more rigorous investigation into the mechanisms underlying LMW HA-induced lymphangiogenesis is clearly warranted.

## 9. Conclusions and Future Perspectives

In the foregoing text, we have summarised the current understanding of how HA and its receptors initiate the entry and trafficking of immune cells in afferent lymphatics based on the recent literature, our own findings and personal insight. Our main focus has been on molecular mechanisms, from the structure and organisation of the immune cell HA glycocalyx, its anchorage by CD44 and engagement with LYVE-1 in transmigratory cups, through its unusual sliding interaction with the receptor that enables leucocytes to transit to the vessel lumen, and finally the signalling pathways downstream of LYVE-1 by which LMW HA may contribute to such trafficking through its promotion of junctional relaxation and new lymphatic vessel proliferation. The ongoing elucidation of these mechanisms has identified LYVE-1 as a docking receptor for DCs and macrophages via their surface HA glycocalyx, and CD44 as the complementary receptor that anchors the glycocalyx and orchestrates lymphatic entry by tuning glycocalyx adhesiveness for LYVE-1. Hence, through mutual interactions with its two key receptors, HMW HA helps facilitate the first committed step in immune cell trafficking between tissues and dLNs, a process vital to the generation of protective immune responses and the resolution of inflammation. Furthermore, LMW HA in inflamed tissues may also contribute by conditioning the lymphatics for immune cell entry through its ability to transduce signals via LYVE-1 for vessel permeability and lymphangiogenesis.

Hence, the LYVE-1 • HA axis is a potential target for limiting the development of unwanted immune responses in the contexts of allograft rejection or early inflammatory disease, where LYVE-1 mediated DC trafficking to dLNs and immune activation are detrimental. This could also include brain and neurological disorders, given that drainage of immune cells, including encephalitogenic T cells, to the deep cervical LNs via meningeal lymphatics, may be critical to the pathology of MS [199]. Clearly, elucidating the crystal structure of the LYVE-1 HA-binding domain should enable the design of novel small molecule inhibitors as well as isolation of new human LYVE-1 function blocking mAbs as therapeutic agents to curtail such responses. As the corollary, during the later resolution phase of tissue injury, when lymphangiogenesis and LYVE-1 • HA-mediated inflammatory cell exit are beneficial, as in MI [161,166] and possibly inflammatory bowel disease [200], it may be beneficial to augment inflammatory cell clearance as a strategy for therapy. Informed by further understanding of the cellular mechanisms regulating LYVE-1 functionality, similar strategies could also be adapted to improve the delivery and efficacy of dermally or mucosally administered vaccines and the HA-based packaging of drugs targeting the lymphatics and downstream dLNs. In terms of blocking immune cell exit, our own preliminary investigations using topically administered mouse LYVE-1 HA-blocking mAbs in an animal model of corneal transplantation indicate such treatments can reduce allograft rejection, and to an extent broadly comparable with that achieved by VEGF-C blockade (Cursiefen, C. Bock, F. Johnson, L.A. and Jackson, D.G., unpublished). Indeed, given the findings that DCs can augment their exit from inflamed tissues by transiting lymphatic collectors via β1 integrin • VCAM-1 adhesion as mentioned earlier [100], it will be interesting to determine whether a combination of LYVE-1 and integrin blockade can further increase effectiveness through synergy.

While we have highlighted the importance of LYVE-1 as the receptor to make the first adhesive contact between migrating immune cell and lymphatic endothelium, the process of vessel entry is clearly more complex and involves the contribution of many other important adhesion receptors, some of which (e.g., ICAM-1) can be present with LYVE-1 in transmigratory cups (Johnson, L.A. and Jackson, D.G., unpublished). Determining how and when these receptors are choreographed in relation to the initial HA • LYVE-1 adhesion events will be an important but challenging goal for the future. Allied to this, it will be interesting to explore whether engagement with LYVE-1 or any of these other receptors by incoming DCs is responsible for transducing signals for the contact-dependent release of CCL21 from lymphatic endothelium that is crucial for directing immune cell entry.

Notwithstanding their involvement in vessel entry, several key questions remain to be addressed as to the roles of HA and its receptors in the subsequent stages of lymphatic trafficking. For example, do adhesive interactions between LYVE-1 and the HA glycocalyx support intraluminal crawling of DCs and T cells directed by downstream-oriented CCL21 chemotactic gradients [96,201] and could they enable such immune cells to reverse transmigrate and re-exit to the tissues under appropriate circumstances? Furthermore, on reaching downstream lymph nodes, do adhesive interactions with the HA glycocalyx mediate the exit of DCs across the floor of the subcapsular sinuses or the transit of immune cells across cortical and medullary sinuses where LYVE-1 is expressed at particularly high levels [202,203,204,205,206]? Lastly, what are the consequences of CD44 co-expression with LYVE-1 in lymph node cortical and medullary sinuses as revealed by single cell RNA sequencing analyses [202,203,206]—do these two functionally distinct HA receptors serve independent or complementary functions in nodal trafficking? Answers to these questions should hopefully be forthcoming in the not too distant future.

## Data Availability

Not applicable.
